# Dietary *Pediastrum boryanum* microalgal extract improves growth, enhances immunity, and regulates immune-related genes in Nile tilapia

**DOI:** 10.1186/s12917-024-04155-z

**Published:** 2024-07-18

**Authors:** Ahmed H. Al-Wakeel, Samia Elbahnaswy, Engy Risha, Eman Zahran

**Affiliations:** 1 Department of Aquatic Animal Medicine, Faculty of Veterinary Medicine, Mansoura University, Mansoura, 35516 Egypt; 2 Department of Clinical Pathology, Faculty of Veterinary Medicine, Mansoura University, Mansoura, 35516 Egypt

**Keywords:** *Pediastrum boryanum*, Growth efficacy, Immunity, Antioxidants, Gene expression, Histomorphology

## Abstract

**Background:**

Identifying alternative sustainable feed sources with high nutritional values is crucial for the future of environmentally and socially responsible aquaculture. In this regard, microalgae have been proven to have positive effects on fish health, which overwhelmed our interest in this study.

**Methods:**

*Pediastrum boryanum* (*P. boryanum*) was incorporated into Nile tilapia feed at concentrations of 0, 0.75, and 1.5 mg/kg, as control, PbExt_0.75_, and PbExt_1.5_ groups to assess its effects on growth and biochemical indices, oxidant/antioxidant activities, immune and stress-related gene expression, and intestinal morphology.

**Results:**

After 8 weeks, fish fed *P. boryanum* supplemented feed exhibited significant increases in final weight, length, condition factor, body weight gain, and specific growth rate, while the spleen-somatic index (SSI) and hepatosomatic index (HSI) showed no significant differences compared to the control group. Dietary *P. boryanum* supplementation also enhanced IgM levels and lysozyme activity, along with no marked effect on markers of liver function enzymes (alanine aminotransferase/ALT and aspartate aminotransferase/AST) or protein status (total protein and albumin). Furthermore, *P. boryanum* addition increased the activity of superoxide dismutase (SOD), catalase (CAT), and reduced glutathione (GSH) enzymes, highlighting its antioxidant potential, whereas malondialdehyde (MDA) concentrations showed no significant differences among the groups. Gene expression analysis revealed that tumor necrosis factor-α (*TNF-α)*, interleukin-10 (*IL-10)*, and transforming growth factor-β1 (*TGF-β1)* expression notably increased in groups fed *P. boryanum* containing feed, while no significant difference was observed in hepatic Heat Shock Protein 70 (*HSP70)* mRNA expression. Histopathological examination revealed no adverse effects of *P. boryanum* supplementation on the liver, spleen, or intestinal tissues. Villous height and villous surface area were notably increased in the high *P. boryanum* supplementation group, suggesting improved intestinal integrity and nutrient absorption.

**Conclusion:**

Dietary *P. boryanum* supplementation can potentially improve growth performance, immune response, antioxidant status, and intestinal health of Nile tilapia, making it a promising candidate for sustainable aquaculture.

## Introduction

The practice of aquaculture, involving the farming of fish, crustaceans, mollusks, and aquatic plants, has been experiencing significant growth driven by the rising demand for aquatic food due to the expanding global population [1]. The volume of global fish production amounted to 186.6 million metric tons in 2023, up from 184.6 million metric tons in 2022 [2]. Aquaculture now provides over 50% of fish for human consumption and is projected to overtake wild-caught fisheries as the primary source of seafood in the coming years [3]. However, the continued expansion and intensification of aquaculture faces sustainability challenges that threaten its long-term viability.

Microalgae, which are microscopic photosynthetic organisms, have emerged as promising feed additives and partial fishmeal substitutes owing to their well-documented nutritional properties [4]. Microalgae are rich sources of proteins, lipids, polysaccharides, pigments, minerals, and vitamins [5–7]. They also contain bioactive compounds such as carotenoids, polyunsaturated fatty acids, and polysaccharides, which can enhance growth, health, and immune responses in farmed aquatic species [8, 9]. Additionally, microalgae production systems have ecological advantages over other feed sources, as microalgae can be cultivated efficiently on non-arable land using non-potable water without the need for fertilizer or pesticide input [10]. Therefore, microalgae are gaining interest for supporting the nutritional, environmental, and economic sustainability of aquaculture.

The green microalga *Pediastrum boryanum* (*P. boryanum*) of the Hydrodictyaceae family is particularly interesting. *P. boryanum* is characterized by flat oval-shaped coenobia comprising 4, 8, 16, or 32 cells surrounded by marginal spines [11]. This ubiquitous freshwater microalga found in lakes and ponds has a rigid cell wall composed of pectin and cellulose [12, 13]. The rigid cell wall confers stability and protects the nutritional content from degradation in the fish digestive system [14]. Valuable nutritional characteristics have spurred interest in exploring *P. boryanum* as a sustainable feed additive to enhance the growth, health, and product quality of farmed animals [15–20]. Therefore, the current study provides evidence for the benefits of dietary *P. boryanum* supplementation on the growth performance and immune responses of key farmed finfish species tilapia (*Oreochromis niloticus*). Overall, this study provides a basis for understanding the diverse benefits conferred by dietary *P. boryanum* and its promising role in supporting sustainable intensification in the aquaculture industry.

## Materials and Methods

### Ethics approval statement

The experiment was conducted according to a protocol involving the use of animals approved by the Mansoura University Animal Care and Use Committee (VM. PhD.23.10.25). All fish handling procedures and regulations followed the ARRIVE guidelines for Animal Care and Use. Furthermore, all relevant organizational and government rules and regulations governing the ethical use of the experimental animals were followed. Written informed consent was obtained from all the participants in this study.

### Diet preparation

The fish feed was prepared in the laboratory of the Department of Nutrition, Faculty of Veterinary Medicine, Mansoura University. Three diets were formulated according to NRC [21], as described in Table 1. An adaptation methodology [22], was used to extract the active components from *P. boryanum,* in accordance with a previous report [23]. *P. boryanum* was acquired from the National Research Center of Cairo, Egypt. Algal powder (100 g) was extracted using 1 L of distilled magnetized water at 70 °C for two hours. Three distinct diets were designed based on the initial diet, incorporating doses of 0, 0.75, and 1.5 mg/kg, and designated as follows. Control, PbExt_0.75_, and PbExt_1.5_, respectively. All ingredients were mixed with gelatin, and water was added until stiff dough was formed. The paste was pelleted into 3-mm-diameter pellets using a meat mincer (ME605131 1600-Watt, Moulinex, Groupe SEB, France). The resulting strands were shadow-dried, broken up, sieved into pellets, and stored in plastic bags at 4 °C until use.

**Table 1:** Ingredients of the Nile tilapia basal diet (air dry basis %)

Ingredient	Control
Yellow Corn	17.87
Soyabean meal	27
Fish meal	16
Wheat bran	32.0
Corn gluten meal	3
Gelatin	2
Sunflower Oil	0.5
Vitamins & mineral premix^a^	0.50
Salt	0.3
Vitamin C	0.1
Antioxidants	0.02
Dicalcium Phosphate	0.5
Methionine	0.21

^a^Trace minerals and vitamin premixes were prepared to cover the levels of microminerals and vitamins in tilapia fish, as recommended by the NRC (1993). Vitamins premix (IU or mg/kg diet); vit. A 5000, Vit. D3 1000, vit. E 20, vit. k3 2, vit. B1 2, vit. B2 5, vit. B6 1.5, vit. B12 0.02, Pantothenic acid 10, Folic acid 1, Biotin 0.15, Niacin 30. Mineral mixture (mg/kg diet); Fe 40, Mn 80, Cu 4, Zn 50, I 0.5, Co 0.2 & Se 0.2

### Experimental design and rearing Fish

A total of 120 apparently healthy Nile tilapias, with an average body weight of 57.5 ± 2.5 g, were selected for this study. The study was conducted as a field trial at a private fish farm in Manzala City, Dakahlia Governorate, Egypt. Fish were randomly assigned to three experimental groups in duplicates (20 fish/hapa). Nile tilapia were allocated to six hapas (200 × 500 × 100 cm^3^, 10 m^3^) for the trial. Water quality parameters were checked on the farm and were maintained within the normal range as follows: 26 -27 °C for water temperature, 6.7 for D.O., 6.5–7.6 for pH, and 0.02–0.15 mg/L for ammonia. Fish were fed at 3% of their body weight throughout the experimental period, divided into two equal rations at 09.00 h and 16.00 h.

### Growth performance assessment

Three fish from each hapa group (*N* = 6) were sampled at the end of the trial. One fish was sampled at a time from each hapa; the three fish were sedated with 30 mg/L of buffered tricane (MS-222®ARGENT) [24], and each was weighed and measured to determine the final body weight (FBW), length, body weight gain (BWG), condition factor (k), and specific growth rate (SGR) [25].

Body weight gain (BWG) = FW − IW, where FW is the final weight and IW is the initial weight.

Condition factor (K) = (W/L^3^) × 100; where: W = weight of fish in grams and L = total length of fish in "cm.”

Specific growth rate (SGR) = 100 × (LN(FW) − LN(IW))/duration (days), where LN = Length, FW = final weight, and IW = initial weight.

All measurements were taken carefully to minimize stress and potential harm to the fish.

### Sample collection

After the trial, fish (six fish per group) were randomly selected and euthanized using buffered tricaine methanesulfonate (E10521-10G, Sigma-Aldrich, UK). Blood samples from the caudal veins was then collected and transferred into sterile tubes and left to clot at room temperature for 4 h before centrifugation at 1198 × *g* for 10 min to collect serum for biochemical and immunological parameter analysis as well as antioxidant status. The fish were then immediately dissected. The liver and spleen were weighed to determine the hepatosomatic index (HSI) and spleen-somatic index (SSI) according to standard AOAC methods [26], respectively, using the following formulae: HSI = Wt of fish liver/fish BWt × 100*,* SSI = Wt of fish spleen/fish BWt × 100*.* A liver sample was placed in RNAlater® (Sigma Aldrich, USA) for the purpose of analyzing immune gene expression. Portions of the liver, spleen, and intestine were preserved in 10% neutral formaldehyde until histopathological examination.

### Evaluation of serum immunological parameters

Serum lysozyme activity was measured using fish lysozyme (LZM) ELIZA kits (MyBioSource, Inc., USA) according to the manufacturer's instructions. The optical density was measured at a wavelength of 450 nm. The concentration of lysozyme in the samples was then determined by comparing the O.D. of the samples to the standard curve, and the lysozyme concentration was reported as µg/mL. In addition, serum IgM levels were determined using fish IgM ELIZA kits (CSBE-12045Fh, CUSABIO BIOTECH CO., Ltd, China), according to the manufacturer's instructions. The optical density was measured at a wavelength of 450 nm. The optical density was measured spectrophotometrically at a wavelength of 450 nm. The concentration of IgM in the samples was then determined by comparing the O.D. of the samples to the standard curve, and the IgM concentration was reported as µg/mL.

### Determination of antioxidant status and oxidative stress markers

Malondialdehyde (MDA) levels and the activities of reduced glutathione (GSH), superoxide dismutase (SOD), and catalase (CAT) were spectrophotometrically measured (6745 UV/Vis. Spectrophotometer) using a colorimetric method. MDA levels, GSH, and SOD activities were measured at 534 nm, 405 nm, and 560 nm, respectively, using diagnostic kits (Biodiagnostics, Egypt), as described elsewhere [27, 28], and were expressed as nmol/L serum. CAT activity was measured using an Elabscience® biochemical assay kit (Elabscience Biotechnology Inc., USA) according to [29], by measuring the decrease in hydrogen peroxide concentration at 240 nm, and was expressed as U/L serum.

### Determination of serum biochemical parameters

Total serum protein and albumin levels were measured spectrophotometrically using test kits (VitroScient, Egypt, Germany). Serum alanine aminotransferase (ALT) and aspartate aminotransferase (AST) levels were estimated using commercial kits (Spinreact, Spain).

### RNA Extraction, Complementary DNA synthesis, and qRT-PCR

Total RNA from each group was extracted from 100 mg of liver tissue in Genzol™ (Geneaid Biotech Ltd, Taiwan) using a manual homogenizer without DNase treatment, followed by dissolving of the pellet in TE buffer (pH 8.0), as described previously [30]. A Nanodrop spectrophotometer (Q5000/Quawell, Massachusetts, USA) was used to determine RNA concentration. Following the manufacturer’s instructions, 1 µg of total RNA was used to synthesize complementary DNA (cDNA) using a TOPscript™ RT DryMIX(dT18) cDNA Synthesis Kit (Enzynomics Co. Ltd., Daejeon, Republic of Korea), according to the manufacturer's protocol. The specific primers used to amplify the selected genes of Nile tilapia with stress marker genes, heat shock protein-70 (*HSP-70*), proinflammatory genes, tumor necrosis factor-alpha (*TNF-α*), anti-inflammatory genes, Transforming Growth Factor-β (*TGF-β1*), and Interleukin-10 (*IL-10*), in addition to *β-actin* as a housekeeping gene, have been previously described [31, 32]. Solg™ 2X Real-Time PCR Smart mix (Including SYBR® Green) was used in conjunction with the QuantStudio1™ Real-Time PCR System (Applied Biosystems™ Thermo Fisher Scientific, USA) to quantitatively analyze gene expression (SolGent Co., Ltd. Yuseong-gu, Daejeon, Korea). The thermocycling conditions were as follows: 95°C for 20 s, followed by 40 cycles of denaturation at 60°C for 40 s, and elongation at 72°C for 30 s. Relative gene expression levels were evaluated in triplicate on template controls using the 2^−ΔΔCT^ formula [33].

### Histopathological examination

The Liver, spleen, and intestine tissue samples were fixed in 10% neutral buffered formalin for 24 h, embedded in paraffin wax, and sectioned at 5 µm. Hematoxylin and Eosin (H&E) was consistently applied to specific slides in accordance with the protocol outlined in [34], to examine their morphology and integrity. Histomorphometric measurements were obtained by examining the stained slides under a light microscope (Olympus CX 31) and capturing images using a connected camera (Olympus DP 21 digital camera) (Olympus Corporation, Tokyo, Japan). This procedure was performed as previously described [35]. The five highest villi per section were identified and selected for measurement. The length from the tip to the bottom and height per section of each villus were determined. The mean values of the measurements were denoted as the villus height per section [36].

### Statistical analysis

Before conducting one-way analysis of variance (ANOVA), Kolmogorov–Smirnov and Levene's tests were used to assess normality and homogeneity, respectively. Analyses were performed using GraphPad® statistics package version 8.4.2. (GraphPad Software, Inc., USA). To examine the differences between the means, Tukey's honestly significant difference test was applied. The significance level was set at *P* < 0.05 (*), *P* < 0.01 (**), and *P* < 0.001 (***). All data are presented as mean ± SEM.

## Results

### Growth performance assessment

**Fig. 1:**
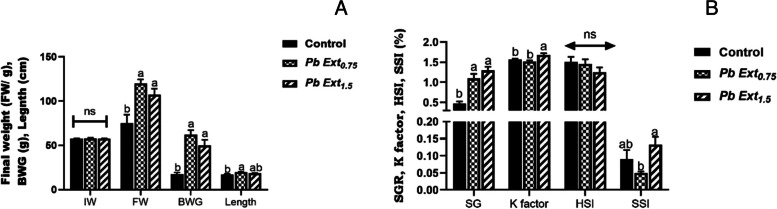
The growth indices, including final weight, length (**A**), K factor, hepatosomatic index (HSI), and spleen-somatic index (SSI) (**B**), of Nile tilapia fed on control or diets supplemented with *P. boryanum* at 0.75 or 1.5 mg/kg. Data were expressed as Mean ± SD. Values with a different letter superscript are significantly different between groups

The bar graphs in Fig. 1 illustrate the growth performance parameters. FW and BWG showed similar trends, where they displayed a significant increase in PbExt_0.75_ and PbExt_1.5_ groups compared to the control group, with no statistical differences between the PbExt-supplemented groups. However, fish length increased significantly in the PbExt_0.75_ group compared to the control, with no variation (*P* > 0.05) compared to PbExt_1.5_. The SGR, K factor, and SSI showed a significant increase in PbExt_1.5_, compared to the control group. The hepatosomatic index (HSI) showed no significant differences across all groups.

### Evaluation of serum immunological parameters

**Fig. 2:**
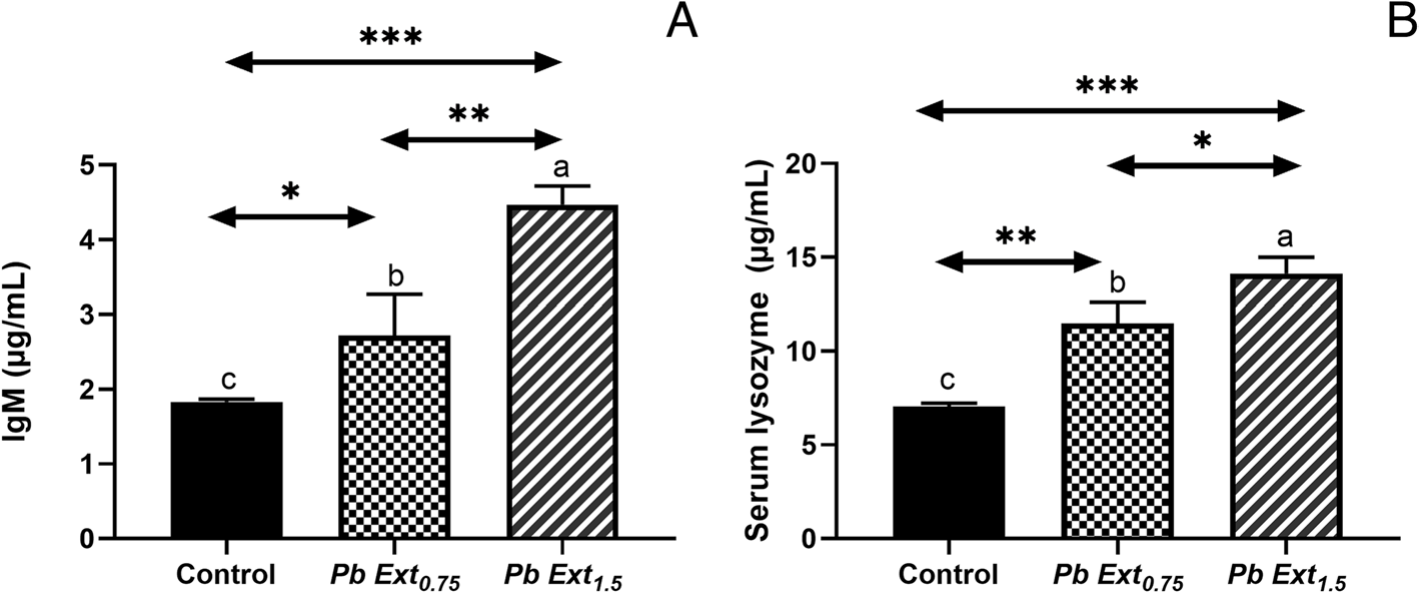
**A** Lysozyme activity and (**B**) IgM level of Nile tilapia fed on control or diets supplemented with *P. boryanum* at 0.75 or 1.5 mg/kg. Data were expressed a Mean ± SD. Values with a different letter superscript are significantly different between groups. Asterisks indicate levels of significance (ANOVA with post hoc Tukey test, *P < 0.05; **P < 0.01; ***P < 0.001)

A notable increase in the activity of lysozyme was observed in both PbExt_0.75_ (*P* < 0.01) and PbExt_1.5_ (*P* < 0.001) groups compared to the control group, with a statistically significant increase in lysozyme (*P* < 0.05) between the fish fed the high level of *P. boryanum* group compared to the other groups (Fig. 2B). The IgM levels in Nile tilapia displayed a noticeable increase in the Nile tilapia-fed PbExt_0.75_ (*P* < 0.05) and PbExt_1.5_.(*P* < 0.001) compared to that in the control group, with a statistically significant increase in both PbExt doses (*P* < 0.01) (Fig. 2A).

### Determination of antioxidant status and oxidative stress markers

**Fig. 3:**
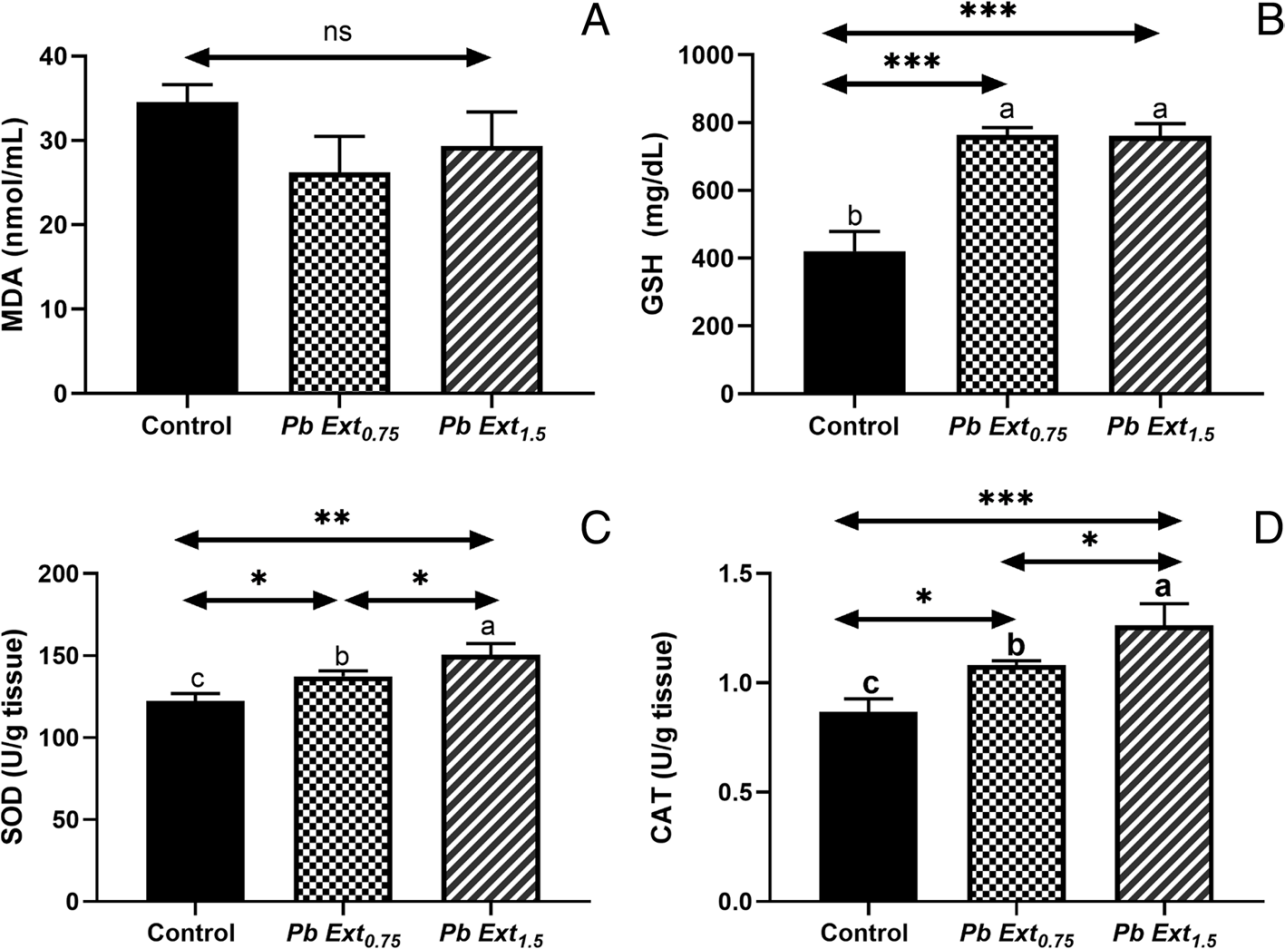
**A** Catalase (CAT), (**B**) glutathione reductase (GSH), and (**C**) Malondialdehyde (MDA) activities of Nile tilapia fed on control or diets supplemented with *P. boryanum* at 0.75 or 1.5 mg/kg. Data were expressed as a Mean ± SD. Values with a different letter superscript are significantly different between groups. Asterisks indicate levels of significance (ANOVA with post hoc Tukey test, *P < 0.05; **P < 0.01; ***P < 0.001)

The oxidant/antioxidant parameters are shown in Fig. 3 A-D. The activities of SOD, CAT, and GSH increased in the groups fed PbExt_0.75_ (SOD, CAT, *P* < 0.05; and GSH, *P* < 0.001) and PbExt_1.5_ (GSH, CAT, *P* < 0.001; and SOD, *P* < 0.01) compared to the control. Only SOD and CAT showed a significant rise in the PbExt_1.5_ (*P* < 0.05) group compared to the PbExt_0.75_ group. Interestingly, the MDA levels (Fig. 3A) were not significantly different among the groups.

### Determination of serum biochemical parameters

**Fig. 4:**
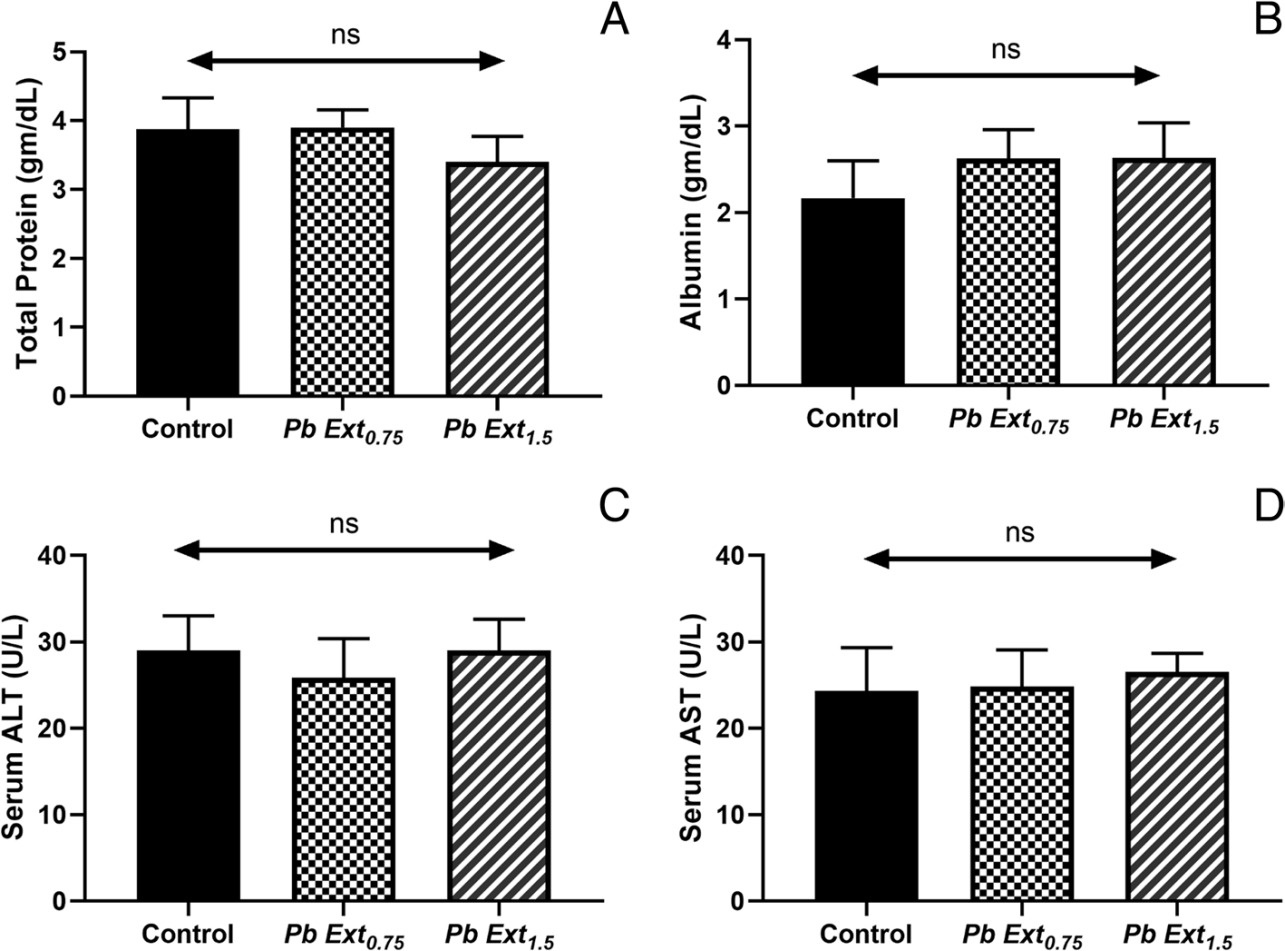
The liver function enzyme activities (**A**) AST, (**B**) ALT, and protein profile [(**C**) total protein and (**D**) Albumin] of Nile tilapia fed on control or diets supplemented with *P. boryanum* at 0.75 or 1.5 mg/kg. Data were expressed as a Mean ± SD. Values with a different letter superscript are significantly different between groups. Asterisks indicate levels of significance (ANOVA with post hoc Tukey test, *P < 0.05; **P < 0.01; ***P < 0.001)

Serum biochemical parameters are presented in Fig. 4 A-D. Liver function enzyme (ALT and AST) and protein profiles (total protein and serum albumin levels) showed no statistical differences between the supplemented and non-supplemented groups.

### Gene expression of Hepatic mRNA level of Nile tilapia

**Fig. 5:**
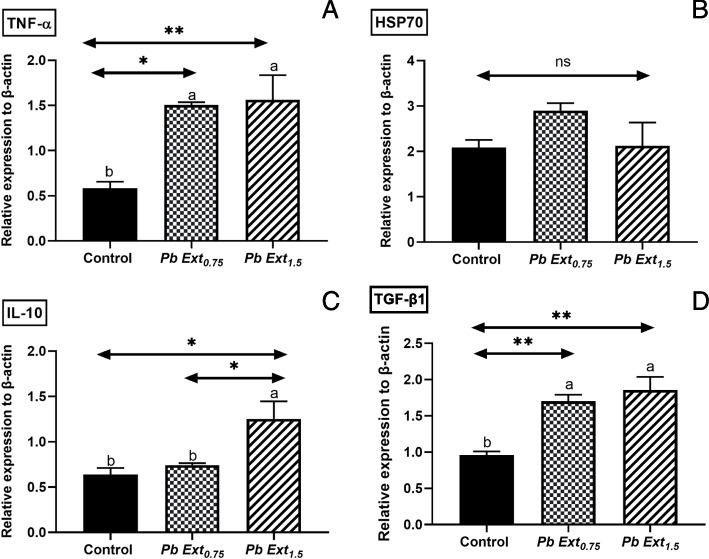
Hepatic gene expression of *TNF-a, TGFβ1*, *IL-10,* and HSP70 in Nile tilapia fed on control or diets supplemented with *P. boryanum* at 0.75 or 1.5 mg/kg. Data were expressed as a Mean ± SD. Values with a different letter superscript or none indicate significance or none between different groups

The bar graphs are shown in Fig. 5. represents the relative expression of proinflammatory and stress genes, including *TNF-α, IL-10, TGF-β1,* and *HSP70*. There was no significant difference in the expression of *HSP70* (Fig. 5B) between the control and supplemented groups. A significant increase in *TNF-α* (Fig. 5A) was observed in both PbExt_0.75_ (*P* < 0.05) and PbExt_1.5_ (*P* < 0.01) groups compared to the control, with no statistical changes between the PbExt supplemented doses. A significant increase was observed in *IL-10* (Fig. 5C) in favor of PbExt_1.5_ compared to both the control (*P* < 0.05) and PbExt_0.75_ (*P* < 0.05) groups, with no statistical significance between the latter. A significant increase was observed in *TGF-β1* (Fig. 5D) in both the PbExt_0.75_ (*P* < 0.01) and PbExt_1.5_ (*P* < 0.01) groups compared to the control group.

### Histopathological examination

**Fig. 6:**
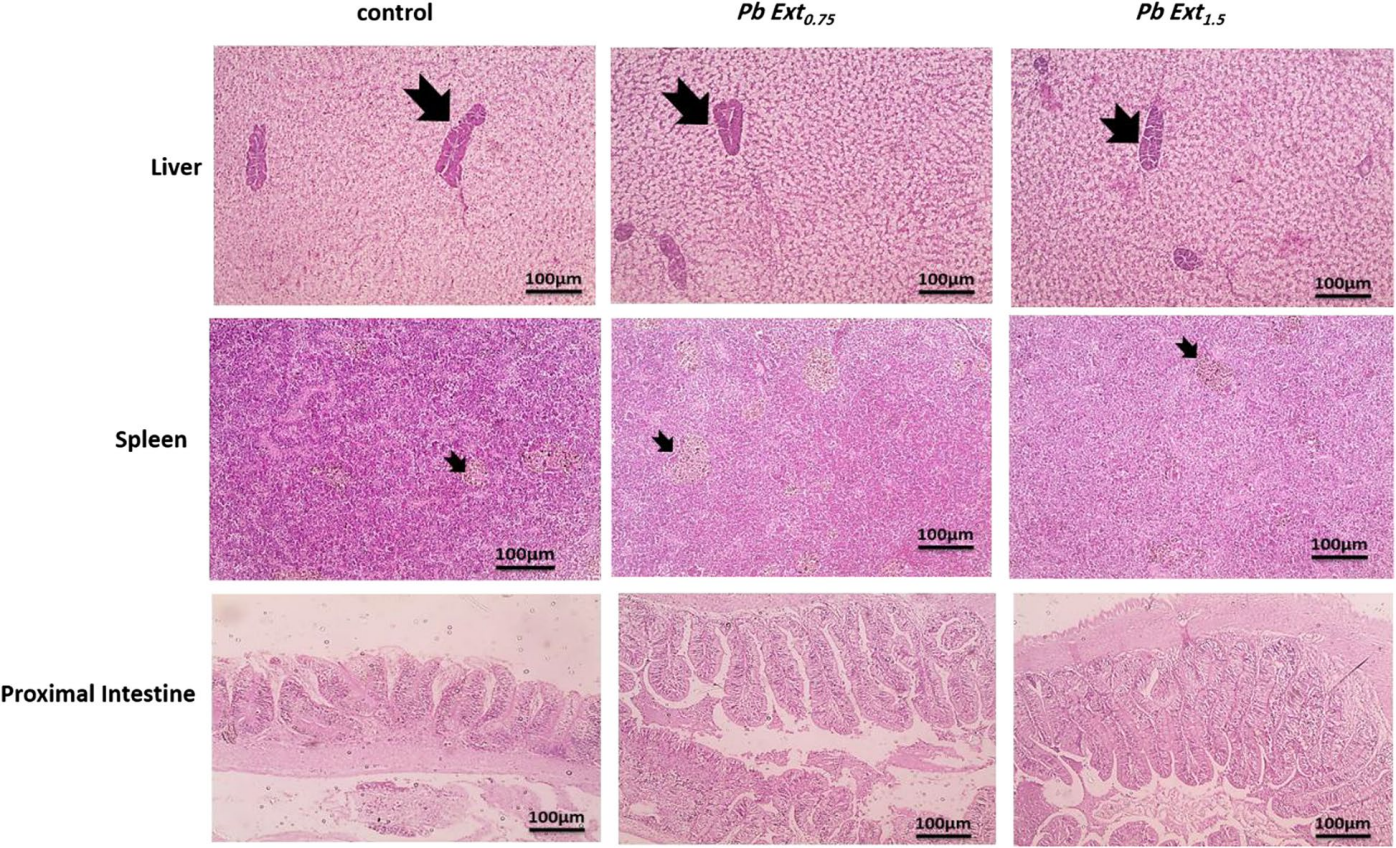
Histopathological examination of the liver, spleen, and proximal intestine of Nile tilapia fed on control or diets supplemented with *P. boryanum* at 0.75 or 1.5 mg/kg

**Fig. 7:**
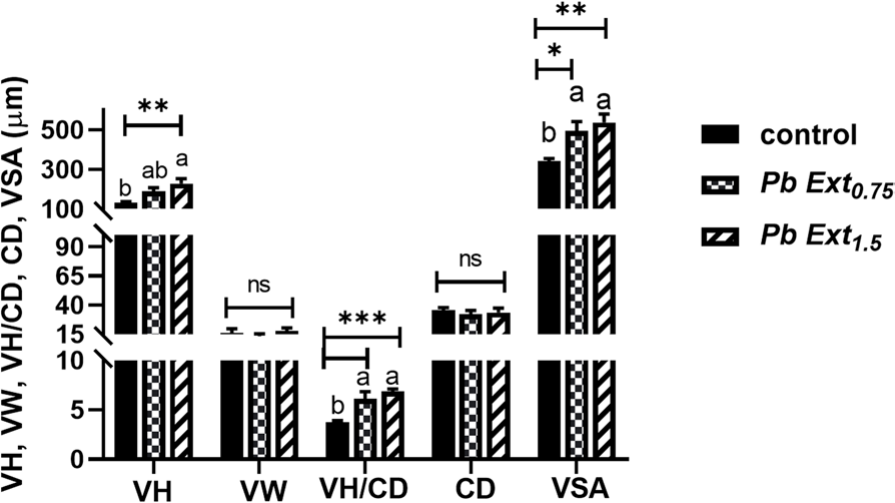
Villous height (VH), villous width (VW), crypt depth (CD), VH/CD ratio, and villous surface area (VSA) in the proximal intestine of Nile tilapia fed on control or diets supplemented with *P. boryanum* at 0.75 or 1.5 mg/kg. Data were expressed as a Mean ± SD. Values with a different letter superscript are significantly different between groups. Asterisks indicate levels of significance (ANOVA with post hoc Tukey test, *P < 0.05; **P < 0.01; ***P < 0.001)

Histopathological examination of the control and supplemented groups revealed a normal histological appearance of hepatocytes, sinusoids, and hepatopancreas in all groups. Similarly, in the spleen, both white and red pulps, including melanomacrophage centers, maintained their histological appearance across all groups. Similarly, in the proximal intestine, there was no observable structural damage in the groups supplemented with the microalgae extract compared with that in the control group (Fig. 6). Interestingly, a marked increase in villous height (VH) was observed in the PbExt_1.5_ group compared to the control. Villous surface area (VSA) and the VH/crypt depth (CD) ratio showed the same trend, whereas VSA increased significantly in both PbExt_0.75_ (*P* < 0.05) and PbExt_1.5_ (*P* < 0.01), and the same VH/CD ratio (*P* < 0.001) compared to the control group, without notable changes between the former. Villous width (VW) and CD did not show any variation (*P* > 0.05) across all groups (Fig. 7).

## Discussion

The freshwater microalga P. boryanum is an excellent fish feed additive because of its exceptional nutritional composition, growth-promoting effects, pigmentation benefits, immunomodulatory properties, and potential for sustainable production [37]. These properties make it a promising fish feed additive. However, there is a lack of research on these comprehensive properties in literature. To the best of our knowledge, this study is the first to investigate comprehensive arrays of parameters to evaluate P. boryanum benefits. Therefore, in our discussion, we will point out similar microalgal extracts used in fish, and we will also cite studies using P. boryanum microalgae in other animal species.

In the present study, *P. boryanum* extract supplementation improved the growth indices at both doses, while displaying no significant changes in the HSI across all groups, indicating a degree of consistency in liver size relative to total body mass. Similar, the addition of mixed microalgal extracts of *Chlorella vulgaris (C. vulgaris)*, *Euglena viridis* (*E. viridis*), and *Spirulina platensis* (*S. platensis*) at 0.01, 0.02% of basal diet exhibited a marked increase in the growth efficacy of *Labeo rohita* fish after 28 days of supplementation [38]. However, no noticeable change was found in the growth of Nile tilapia juveniles fed diets containing an extract obtained from the green alga *Ulva clathrata* at higher doses (0.5–1%) [39]. These discrepancies could be related to the type of extract used, the dose of supplementation, the fish spp and its life stage, and the rearing conditions, either in indoor tanks or farm ponds. Another study conducted by Ahmed et al. [40], reported that rabbits fed a diet enriched with 10 ml/kg *P. boryanum* extract exhibited notable enhancements in final body weight, daily weight gain, and feed conversion ratio. These findings could be attributed to the nutritional composition of *P. boryanum*, as it is a good source of proteins, essential fatty acids, minerals, carotenoids, chlorophylls, and phenolic compounds with antioxidant properties [15–19]. For instance, *P. boryanum* contains 45–60% protein, 9–14% lipids including linoleic and α-linolenic acids, and carotenoids such as violaxanthin, lutein, and β-carotene at concentrations ranging from 4.4 to 29.2 mg/g dry weight [15, 18, 20]. Therefore, its high protein content reflects an increase in growth performance, and the rigid cell wall of *P. boryanum* confers stability and protects its nutritional content from degradation in the fish digestive system [14]. These findings highlight the potential of *P. boryanum* as a dietary supplement that influences the growth performance of Nile tilapia.

Lysozyme activity and IgM levels were significantly elevated in the PbExt_0.75_ and PbExt_1.5_ groups compared to the control. Coincided with our findings, rohu (*Labeo rohita*) fed with a mixed algal extract (*C. vulgaris*, *E. viridis*, and *S. platensis)* at 0.01 and 0.02% of the basal diet showed significantly higher activity of lysozyme after 28 days of experimental trial [38]. Also, It has been reported that dietary inclusion of *Ulva clathrata* (*U. clathrata*) extract at a lower level of 0.1% of the diet displayed a noticeable amelioration in the innate immunity parameters, such as lysozyme and complement activities, after 60 days of feeding [39]. Furthermore, the dietary addition of 0.5% of red algal extract (*Laurencia caspica*) to the Nile tilapia showed a significantly increased in lysozyme and IgM activities after 50 d [41]. It has been suggested that *P. boryanum* supplementation elicits B lymphocytes owing to its polysaccharide content, thereby augmenting both innate and adaptive immune responses [13]. Additionally, arachidonic acid, a precursor of biologically active prostaglandins and leukotrienes, promotes leukocyte chemotaxis, reactive oxygen species formation, and other pro-inflammatory effects [42].

The results of this study provide intriguing insights into the effects of *P. boryanum* microalgal extract on oxidative stress markers and antioxidant status in Nile tilapia, in which antioxidant enzyme activities were enhanced without oxidative stress. Our results are consistent with Khanzadeh et al. [41], who reported a significant increase in SOD and CAT after the dietary addition of 0.5% of red algal extract (*Laurencia caspica*) to the Nile tilapia for 50 days. Similarly, Abdel-Latif and Khalil [43], reported that dietary supplementation with *S. platensis* (2.5%) for eight weeks significantly enhanced the activities of CAT, SOD, GSH-Px in Nile tilapia. Furthermore, myeloperoxidase activity displayed no significant changes in *Labeo rohita* fed mixed algal extract at 0.1 and 0.2% of basal diet [38], and in hybrid red tilapia fed *Haematococcus pluvialis*- microalgal extract at 0.5. 1, and 1.5 mg/kg for 60 days [44], which was attributed to the biological constituents of *P. boryanum* microalga, such as carotenoids and canthaxanthin, which play a remarkable role as antioxidant compounds and free radical scavengers. Furthermore, polysaccharide content enhances antioxidant defenses [45]. *P. boryanum* biosynthesizes and accumulates high levels of carotenoids, chlorophylls, and phenolic compounds with antioxidant properties [15–19].

There were no adverse effects on liver function enzymes and protein profiles, as indicated by the lack of statistical changes. Previous studies showed similar results, finding that *Labeo rohita* fed a 0.08% diet of mixed algae extract (*C. vulgaris*, *E. viridis*, and *S. platensis)* for 28 days had no significant albumin-globulin ratio than the control group [38]. Similarly, protein profile, ALT and AST levels did not change in Nile tilapia fed 0.5% of red algal extract (*Laurencia caspica*) for 50 days [41]. The same findings were observed in Gibel carp upon feeding algal extract at (*Ulva lactuca* and *Solieria chordalis*) 0.2% [46]. Other studies concerning *P. boryanum* in other animal species revealed same effects as well, Ahmed et al. [40], showed no statistical changes in liver enzyme levels (ALT and AST) or plasma albumin in New Zealand white rabbits fed *Pediastrum* spp. Silva et al. [13] showed that the administration of various *P. boryanum* extracts did not alter the serum levels of the liver enzymes ALT and AST in rats. Fonseca et al. [47] reported no significant increases in liver ALT and AST enzymes in mice orally administered 300 mg/kg or 2000 mg/kg freeze-dried *P. boryanum* biomass suspended in carboxymethyl cellulose. These findings suggested that there were no adverse effects on liver function or disruption of protein homeostasis. The lack of significant changes in these biochemical parameters coupled with the MDA, HSP70 expression level, and histopathology results (see below) support its safety on fish health, and Fonseca et al. [47] classified P. boryanum as having minimal toxicity or security. Our findings contribute to the overall understanding of the safety and physiological compatibility of *P. boryanum* supplementation in Nile tilapia, encouraging its potential application in aquaculture, without apparent negative effects on liver health or protein metabolism.

*P. boryanum* dietary supplementation showed no significant hepatic gene expression of *HSP70*, whereas *TNF-α*, *IL-10*, and *TGF-β1* expression was significantly increased in groups fed with *P. boryanum*. Tumor Necrosis Factor-alpha (*TNF-α*) functions as a pro-inflammatory cytokine and serves as a valuable prognostic indicator for immune responses in fish [48], whereas Heat Shock Protein 70 (*HSP70*) functions as a molecular chaperone, safeguarding fish cells against environmental stressors [49]. Interleukin-10 (*IL-10*) acts as an anti-inflammatory cytokine, preventing tissue damage and chronic inflammation by reducing unnecessary T-cell responses to microbial infections [50]. Transforming Growth Factor Beta 1 (*TGF-β1*), a versatile cytokine, orchestrates cellular functions such as growth, differentiation, and immune responses and crucially contributes to tissue homeostasis and adaptive reactions during injury or inflammation [50]. The increase in *TNF-α* gene expression upon *P. boryanum* extract administration showed the immunostimulatory property of the microalga as the polysaccharide content from the microalgal extract stimulated phagocytes to produce cytokines [39]. Similarly, dietary inclusion of *Nannochloropsis gaditana* microalgal extract at 10 ml/kg of diet in zebra fish (*Danio rerio*) [51], and *Haematococcus pluvialis* microalgal extract at 0.5. 1, and 1.5 mg/kg in hybrid red tilapia fed for 60 days [44], and mixed algal extract at 0.01 and 0.02% of basal diet in *Labeo rohita* for 28 days displayed cytokine expression by upregulating the expression of TNF-α and *IL-10* genes. Similarly, increased transcripts *TNF-α* mRNAs were significantly modified in the *senegalese sole* post-larvae administered with crude extract of *N. gaditana* (0.2 mg dry mass equivalent mL^−1^ sea water) [52]. To date, there are no reports on the effect of the microalgal extract on the expression of hepatic cytokines, so the upregulation of both pro-and anti-inflammatory cytokines in Nile tilapia-fed *P. boryanum* diets might be an effort to balance the excess production of anti-inflammatory cytokines, namely *IL-10, TGF-β1* which can compromise the host's ability to remove microorganisms through suppression of immune cell function, proposing immunomodulatory effects [51].

Our findings shed light on the antioxidative effect of the microalga *P. boryanum,* which reflected the stable expression of *HSP70* among groups, together with the MDA results herein, confirming that no stresses were revealed upon *P. boryanum* feeding on Nile tilapia. Furthermore, bioactive compounds in microalgae, such as flavonoids, polysaccharides, and polyunsaturated fatty acids (arachidonic acid), have been associated with anti-inflammatory activity by reducing neutrophil infiltration and modulating proinflammatory cytokines [13], thus maintaining fish health and homeostasis [45].

Our previous analyses herein were histopathologically evident, where no alteration in tissues architecture had been found, besides, favorable histomorphometric indices, particularly the VH, VH/CD, and VSA were observed, which positively influenced nutrient absorption with subsequent growth improvement. Similarly, Zebrafish juveniles supplemented with 10 g/kg of *Nannochloropsis gaditana* showed no inflammatory changes in intestinal histopathological examination and positively affected their morphometry [51]. In the same context, found no negative influences of feeding 1.2% microalgae (*Schizochytrium* sp.) in Nile tilapia [53]. In the same context, hybrid red tilapia fed *Haematococcus pluvialis* microalgal extract at 1 g/kg for 60 days enhanced the intestinal villi length and width, and no histopathological lesions were induced in liver or intestine fed the microalgal extract [44]. The data obtained as mentioned earlier are due to the bioactive constituents of microalgae, which contribute to the suppression of oxidative damage and inflammatory changes, thus maintaining fish growth, immune status, and overall health.

## Conclusion

In conclusion, this study provides evidence that *P. boryanum* microalgal extract has beneficial effects as a feed additive for Nile tilapia (*Oreochromis niloticus*). Fish fed *P. boryanum* extract demonstrated improved growth performance and innate immune and antioxidant status, and induced no oxidative stress or inflammatory changes. Furthermore, *P. boryanum* maintains intestinal histomorphology and health. Overall, this study provides a strong basis for utilizing *P. boryanum* microalgal extract to develop efficient and sustainable feed that can enhance the productivity and quality of farmed tilapia.

## Data Availability

Data is provided within the manuscript.

## Abbreviations

BWG: Body weight gain
FW: Final weight
IW: Initial weight
K: Condition factor
SGR: Specific growth rate
MSS-222: Tricaine methanesulfonate
HSI: Hepatosomatic index
SSI: Spleenosomatic index
LZM: Lysozyme
MDA: Malondialdehyde
GSH: Reduced glutathione
SOD: Superoxide dismutase
CAT: Catalase
ALT: Alanine aminotransferase
AST: Aspartate aminotransferase
AOAC: Association of Official Analytical Chemists
DW: Distilled water
NRC: National Research Council
*P. boryanum*: *Pediastrum boryanum*
ROS: Reactive oxygen species
*TNF-α*: Tumor necrosis factor-*α*
*HSP70*: *Heat Shock Protein 70*
*TGF-β1*: Transforming Growth Factor-β
*IL-10*: Interleukin-10
